# Intensity Modulated Radiotherapy (IMRT) With Carbon Ion Boost in the Multimodal Treatment of Salivary Duct Carcinoma

**DOI:** 10.3389/fonc.2019.01420

**Published:** 2019-12-20

**Authors:** Sebastian Adeberg, Paul Windisch, Felix Ehret, Melissa Baur, Sati Akbaba, Thomas Held, Denise Bernhardt, Matthias F. Haefner, Juergen Krauss, Steffen Kargus, Christian Freudlsperger, Peter Plinkert, Christa Flechtenmacher, Klaus Herfarth, Juergen Debus, Stefan Rieken

**Affiliations:** ^1^Department of Radiation Oncology, Heidelberg University Hospital, Heidelberg, Germany; ^2^Heidelberg Institute for Radiation Oncology, Heidelberg, Germany; ^3^Clinical Cooperation Unit Radiation Oncology, German Cancer Research Center (DKFZ), Heidelberg, Germany; ^4^Heidelberg Ion-Beam Therapy Center, Heidelberg, Germany; ^5^National Center for Tumor Diseases, Heidelberg, Germany; ^6^Department of Oral and Maxillofacial Surgery, Heidelberg University Hospital, Heidelberg, Germany; ^7^Department of Otorhinolaryngology, Heidelberg University Hospital, Heidelberg, Germany; ^8^Department of Pathology, Heidelberg University Hospital, Heidelberg, Germany

**Keywords:** radiation therapy, bimodal radiotherapy, carbon ion radiotherapy, toxicity, salivary gland, intensity-modulated radiotherapy

## Abstract

**Background:** To assess outcomes and treatment related toxicity following intensity-modulated radiotherapy (IMRT) and a Carbon Ion Radiotherapy (CIRT) boost for salivary duct carcinoma (SDC).

**Methods:** Twenty-eight consecutive patients with SDC who underwent a postoperative (82%) or definitive (18%) radiation therapy between 2010 and 2017 were assessed in this retrospective single-center analysis. CIRT boost was delivered with median 18 Gy(RBE) in 6 daily fractions, followed by an TomoTherapy^®^-based IMRT (median 54 Gy in 27 daily fractions). Treatment-related acute toxicity was assessed according to CTCAE Version 4.

**Results:** Tumors were most commonly located in the major salivary glands (*n* = 25; 89%); 23 patients (82%) received previous surgery (R0: 30%; R1: 57%; R2: 4%; RX: 19%). Median follow-up was 30 months. Four patients (14%) experienced a local relapse and 3 (11%) developed locoregional recurrence. The two-year local control (LC) and locoregional control (LRC) was 96 and 93%, respectively. Median disease-free survival (DFS) was 27 months, metastasis-free survival (MFS) was 69 months, and overall survival (OS) was 93 months. Acute grade 3 toxicity occurred in 11 patients (mucositis, dermatitis, xerostomia; *n* = 2 each (7%) were the most common) and 2 osteonecroses of the mandibular (grade 3) occurred. No patients experienced grade ≥4 toxicities.

**Conclusions:** Multimodal therapy approaches with surgery followed by IMRT and CIRT boost for SDC leads to good local and locoregional disease control. However, the frequent occurrence of distant metastases limits the prognosis and requires optimization of adjuvant systemic therapies.

## Introduction

SDC were first described by Kleinsasser and colleagues in 1968 as a separate group of “adenocarcinomas” of the salivary gland displaying a histopathological resemblance to ductal carcinoma of the breast; the World Health Organization recognized these tumors as a distinct tumor entity in 1991 (1, 2). Since then, SDC refer to rare but highly aggressive tumors originating from the ductal epithelium of major salivary glands (3). Malignant salivary gland tumors (MSGT) have an estimated annual incidence rate of 1 to 1.2 per 100,000 (4). SDC account for approximately 1% to 3% of all malignant salivary gland tumors and mostly occur during the fifth to seventh decade of life; men are predominantly affected (4–14). Current treatment options include surgery, systemic therapy, radiation and targeted therapy. Surgical management typically involves complete surgical resection and lymphadenectomy; depending on the tumor localization and stage, this can include parotidectomy, submandibular excision, ipsilateral, and contralateral neck dissection. Owing to its rarity and often poor response, the role of systemic therapies has only been investigated in case series and small clinical trials (15, 16).

Despite the advancements in surgery, systemic therapy and radiotherapy, the prognosis of SDC remains meager (6). Despite showing trends toward improved local control, especially the role of adjuvant radiotherapy remains unclear (6, 17–20). These tumors are currently treated in analogy to other MSGT. In the adjuvant setting, radiotherapy plays a role in patients with higher risk disease, e.g., perineural invasion (PNI), R+, T3/4 tumors. Additionally, definitive radiotherapy is a valuable alternative for unresectable cases. In the adjuvant setting, dose response relationships were described for both LRC (21) and LC (22) in MSGTs. Here, high-linear energy transfer (LET) radiation therapy (e.g., with charged particles such as carbon ions) can lead to improved tumor control rates in other head/neck malignancies as compared to standard photon therapy (22, 23). The objective of this retrospective, single-institutional study is to provide further clinical data and prognostic factors regarding intensity modulated radiotherapy (IMRT) with carbon ion radiotherapy (CIRT) in patients with SDC.

## Materials and Methods

### Patient Population

Patient records, surgical reports, histological work-up, and radiotherapy treatment plans of patients with SDC who underwent IMRT-CIRT between August 2010 and November 2017 in the Department of Radiation Oncology, University Hospital and at the Heidelberg Ion-Beam Therapy Center (HIT) were evaluated retrospectively. A subset of patients (18%) with locoregional advanced disease or unresectable received a primary radiotherapy.

### Radiation Therapy

Treatment planning was performed using native and contrast enhanced CT/MRI. Patients were immobilized with individualized thermoplastic head masks. Technical details of CIRT are described elsewhere (24, 25). Treatment planning for CIRT was performed using Syngo PT Planning, Version 13 (Siemens, Erlangen, Germany) and TomoTherapy^®^-Planning Station (Accuray, Sunnyvale, CA, USA) for photon radiotherapy planning. Patients were treated with a fixed horizontal beam/gantry for CIRT utilizing 1-2 coplanar/non-coplanar beams.

**Figure 1:**
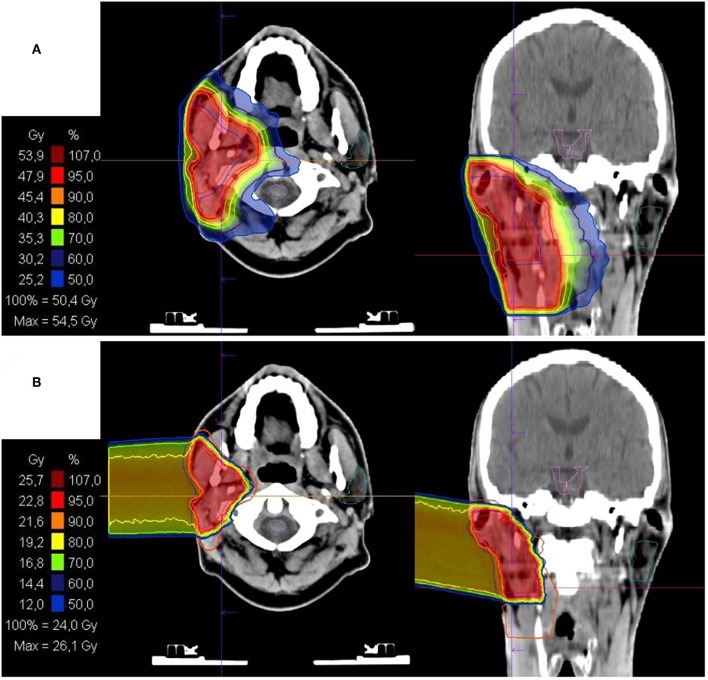
Bimodal radiotherapy treatment plan: **(A)** intensity modulated radiotherapy (IMRT) base plan with 50Gy in 2Gy/fraction and **(B)** active raster-scanning carbon ion radiotherapy (CIRT) boost plan with 28Gy (RBE) in 3Gy (RBE)/fraction. Treatment was delivered as a definite radiotherapy in a patient with a recurrent rcT2 rcN2b cM0 salivary duct carcinoma (SDC) of the right parotid gland. CIRT was applied with one lateral beam. Histopathological work up revealed Her2neu and androgen receptor (AR) positivity. Therefore, the patient received an adjuvant therapy with trastuzumab and bicalutamide.

All patients received combined IMRT and CIRT. The base plan was performed using a helical intensity-modulated radiotherapy (IMRT) with daily image guidance (TomoTherapy^®^, Accuray, Sunnyvale, CA, USA), with 5 daily fractions per week (Figure 1).

### Target Volume Delineation and Dose Prescription

Target delineation was based on native/contrast enhanced CT scans fused with contrast-enhanced MRI. Two clinical target volumes (CTV1/CTV2) were outlined. CTV2 comprised the macroscopic tumor and/or tumor bed. CTV1 included CTV2 as well as local growth patterns; ipsilateral nodal levels II-III were included into the CTV1 as well. A 3 mm margin was added to the CTVs to generate the planning target volumes (PTVs).

Organs at risk such as the spinal cord, contralateral parotid gland, temporomandibular joints, and the optic system were constrained per QUANTEC data (26). CTV1 received a median dose of 54 Gy (range: 50–56 Gy) in 2 Gy daily doses (1.8 Gy to 2.0 Gy) (median equivalent dose in 2 Gy fractions (EQD2) of 50 Gy). CTV1 was to be covered by the 90% isodose line. A sequential CIRT boost was applied to the CTV2 utilizing an intensity-controlled active raster-scanning technique, in 3 Gy [relative biological effectiveness (RBE)] fractional doses up to a median combined EQD2 (Equivalent dose in 2Gy fractions) = $\text{D}\frac{\left[\text{d}+\left(\alpha /\beta \right)\right]}{\left[2+\left(\alpha /\beta \right)\right]}$ (where, D = total dose given in Gy, d = dose per fraction in Gy, and α/β = is assumed to be 2) of 78.5 Gy (range 78.5–80 Gy). We aimed for the CTV2 to be covered by the 95% isodose line. The following equation was used to calculate biologically effective dose (BED) = $nd\left(1+\frac{d}{\alpha /\beta }\right)$ (where n is the number of fractions, d is fractional dose (in Gy), and α/β is assumed to be 2). The total CIRT dose of 18–24 Gy (RBE) corresponds to a BED of 45–60 Gy.

### Follow Up

Patients were monitored on treatment weekly with toxicity assessments (CTCAE classification v.4). Follow-up included a clinical examination by an otorhinolaryngologist and contrast enhanced MR-imaging of the head and neck every 3 months for the first 2 years after radiotherapy, every 6 months until the fifth year after treatment, and annually thereafter. Staging CTs were performed yearly to exclude distant metastases.

### Survival, Local, and Locoregional Control

Local control (LC) and locoregional control (LRC) rates were calculated by Kaplan-Meier estimates, from the start of therapy until local tumor progression/death and/or nodal failure. Patients without tumor progression and patients lost to follow-up were censored.

Metastasis-free survival (MFS) and disease-free survival (DFS) were calculated by Kaplan-Meier estimates, defined from the start of therapy until distant metastases occurred or progression/relapse at any location, respectively. Patients without events and those lost to follow-up were censored.

Overall Survival (OS) was calculated by Kaplan-Meier estimates, from the start of therapy until death or last contact (alive subjects were censored).

### Data Analysis

The log-rank test for univariate analysis was performed to assess prognostic factors for survival. Statistical analyses were performed using SigmaPlot™ (Systat Software GmbH, Germany) software, and a *p*-value of <0.05 was considered statistically significant.

## Results

### Treatment Setup and Tumor Characteristics

Detailed patient characteristics are depicted in Table 1. Overall 28 consecutive patients were included with a median age of 69 years (range 41–83 years). 79% (*n* = 22) of tumors were localized in the parotid gland.

**Table 1:** Clinical characteristics of the study cohort (*n* = 28).

**Parameter**	**Median (range or %)**
Age (years)	69 (41–83)
KPS	90 (60–100)
**Gender**	
Male	25 (89%)
Female	3 (11%)
**Primary location**	
Parotid gland	22 (79%)
Submandibular gland	2 (7%)
Minor salivary glands	2 (7%)
Sublingual gland	1 (4%)
Lacrimal gland	1 (4%)
**T classification**	
T1	2 (7%)
T2	3 (11%)
T3	10 (36%)
T4	13 (46%)
**N classification**	
N0	9 (32%)
N1	2 (7%)
N2	14 (50%)
N3	2 (7%)
NX	1 (4%)
**M classification**	
M1	1 (4%)
**PNI**	
Yes	14 (50%)
No	6 (21%)
n.e.	8 (29%)
**LV**	
Yes	11 (39%)
No	10 (36%)
n.e.	7 (37%)
**Resection status**	
R0	7 (30%)
R1	13 (57%)
R2	1 (4%)
Rx	2 (9%)
**Her2neu**	
Positive	14 (50%)
Negative	10 (36%)
n.e.	4 (14%)

*PNI, perineural invasion; LV, lymphovascular invasion; n.e., not examined; KPS, Karnofsky performance status*.

*Numbers may not add to 100% owing to rounding and multiple categorizations for single specimens*.

Twenty-three patients (82%) underwent surgical resection (parotidectomy, *n* = 17; mastoidectomy, *n* = 4; modified neck dissection, *n* = 20) followed by postoperative radiotherapy. Five patients (18%) received a definitive radiotherapy. Median clinical target volume (CTV) and planning target volume (PTV) dimension of the CIRT boost was 120cc (range 36–639cc) and 187cc (range 63–817cc). Median time interval between surgery and commencement of radiotherapy was 57 days (range: 30–135 days). Complete surgical resection (R0) was achieved in 7 patients (30%). The majority of tumors initially presented at advanced stages (T3, *n* = 10, 36% and T4, *n* = 13, 46%) and with lymph node involvement (N1, *n* = 2, 7%; N2, *n* = 14, 50%; N3, *n* = 2, 7%). PNI (50%) and lymphovascular invasion (LVI) (39%) was common. Her2neu was positive in 14/24 tested patients (58%). tumor tissues were positive for androgen receptors Most (19/23, 83%). Adjuvant systemic therapy with the antiandrogen agent bicalutamide was delivered to 7 patients and bicalutamide with trastuzumab in 5 patients.

### Survival and Local Control

**Figure 2:**
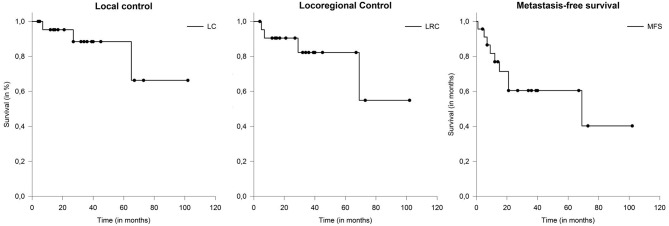
The actuarial 2-year LC and LRC was 96 and 93%, respectively. Metastasis-free survival (MFS) in patients with salivary duct carcinoma (SDC) 69 months.

After a median follow-up of 30 months (range: 8–109 months), 17 patients (61%) were still alive. Local tumor progression was observed in 3 patients (11%) and nodal failure was observed in 4 patients (14%). Median LC and LRC were not reached (Figure 2). The actuarial 2-year LC and LRC was 96 and 93%, respectively. Distant metastases occurred in 9 patients (32%) over the course of disease. Median metastasis-free survival (MFS) was 69 months (range: 4–102).

**Figure 3:**
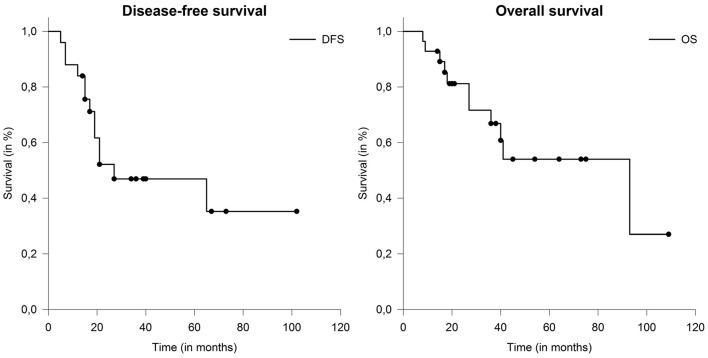
Median disease-free survival (DFS) and overall survival (OS) in patients with salivary duct carcinoma (SDC) were 27 and 93 months.

The most frequent location of distant metastases was pulmonary (21%) and osseous (14%) areas. Metachronous distant metastases occurred in 4 patients (21%). In one patient, preexisting bipulmonary metastases were progressive. Overall, the median disease-free survival (DFS) was 27 months (range: 4–107 months). Median overall survival (OS) was 93 months (range: 9–109 months) (Figure 3). Five cases who underwent definitive radiotherapy did not experience a local relapse during follow-up, but 3 of 5 experienced distant metastases after 6, 7, and 15 months. Patient and tumor characteristics between definitive and postoperative treated patients did not differ significantly. Here median DFS (*p* = 0.23) and OS (*p* = 0.58) did not show statistical differences, even though patient cohorts were rather small for comparison.

### Prognostic Factors

**Figure 4:**
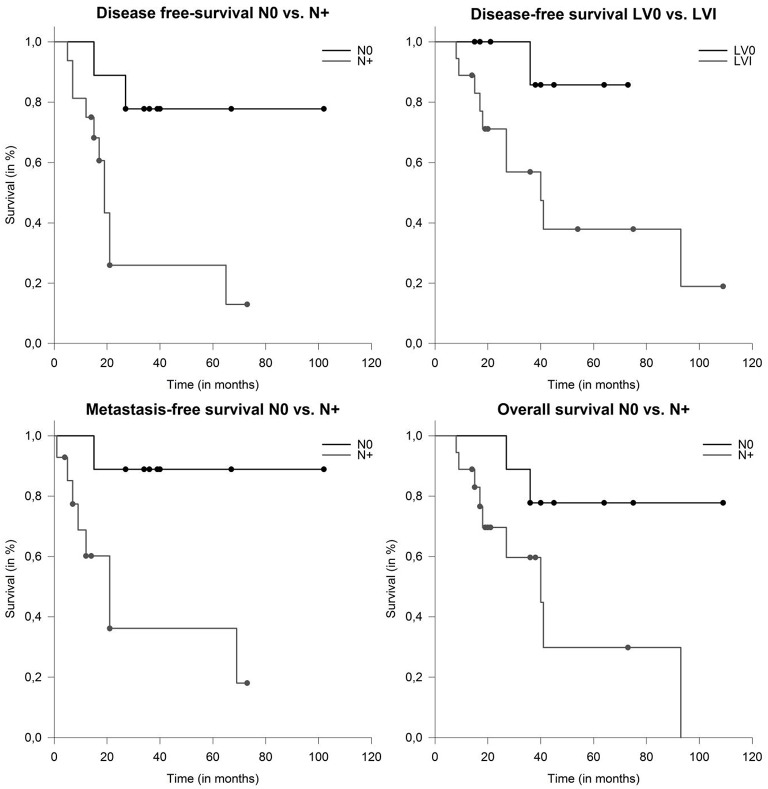
Median disease-free survival (DFS) depending on nodal involvement (*p* = 0.009) and lymphovascular involvement (*p* = 0.045). Metastasis-free survival and overall survival (OS) in patients with salivary duct carcinoma (SDC) depending on nodal involvement (*p* = 0.02 and *p* = 0.039).

On univariate analysis larger CTV and PTV dimension of the CIRT boost (both continuous variates) were prognostic for impaired DFS (*p* = 0.026 and *p* = 0.003), MFS (*p* = 0.006 and *p* = 0.007) and OS (*p* = 0.005 and *p* = 0.005). Nodal involvement was prognostic for poor DFS (*p* = 0.022), MFS (*p* = 0.044) (Figure 4) and showed a trend toward impaired OS (*p* = 0.059). LVI was associated with impaired DFS (*p* = 0.045) and OS (*p* = 0.041) (Figure 4). Other known prognostic factors like T-stage, Her2neu, PNI, age, adjuvant systemic therapies and resection status did not show a correlation with any endpoint.

### Treatment Related Toxicities

Acute grade 1 and 2 fatigue, mucositis, xerostomia, and dermatitis were commonly observed in the study cohort. Ten acute grade 3 toxicities [two each (7%) of mucositis, dermatitis, xerostomia; and one each (3%) of dysphagia, odynophagia, dysgeusia, nausea/emesis] occurred in 7 patients (25%). In 11 patients (58%) a preexisting facial palsy remained stable during/after radiotherapy. Regarding late adverse event, two osteonecroses of the mandibular jaw occurred 24 and 32 months after radiotherapy. In one patient a surgical intervention was necessary (grade 3) and led to satisfactory long-term results. Overall, no acute or late grade ≥4 toxicities were reported.

## Discussion

Although surgery combined with IMRT and CIRT resulted in appropriate LC and LRC, prognosis of patients with SDC is limited by the high rate of distant metastases underlined by a poor MFS in our cohort.

However, even when definitively treated, SDCs are linked to a meager prognosis with most of the patients dying within 5 years of diagnosis (7, 9, 14, 39, 40, 44). High rates of local recurrence (15–55%) and distant metastases (33–62%) account for the worse outcome (33). Local approaches should thus include radical surgical resection e.g., with parotidectomy and neck dissection whenever possible.

Postoperative radiotherapy in SDC is mainly performed as extrapolations from head and neck tumors including MSGT. However, larger series that focus on the predictors and outcome after radiotherapy are lacking. Smaller series report of 5-year LC, DFS, and OS rates of 67, 45, and 47% after adjuvant radiotherapy with a median photon dose of 60Gy. The authors advise including nerves tracked to the skull base if PNI is presented (19). The addition of radiotherapy can reduce local recurrence rates from approximately 30 to 10% without impacting OS (21). Summarizing various single institution experiences, LC rates were encouraging after surgery and postoperative radiotherapy (17, 20).

Overall a benefit for radiotherapy dose escalation for MSGTs has been shown, for instance in adenoid cystic carcinoma (ACC) (45, 46). The data for the subgroup of SDC, however, is unclear. The current study presents the first data of advanced radiation techniques with IMRT and high-LET CIRT. With regard to local control in these relatively radioresistant tumors, high-LET radiotherapy seems to be beneficial. In this context, the biophysical advantages with its steep dose-gradient and superior relative biological effectiveness (RBE) allow for safer dose-escalation, like previously described in other tumor entities of the head and neck (47–52) Furthermore the high physical conformity, compared to photons and decreased lateral scattering as with other particles lead to decreased dose to normal tissue (53). This potentially translates into improved local control by means of safer dose escalation combined with improved sparing of organs at risk. Despite negative prognostic factors in the majority of patients in our cohort, LC and LRC rates of 96 and 93% after 2 years were favorable compared to other reports in the literature (7, 9, 15, 36, 43). Our experiences of relatively low DFS and MFS are supported by previous series (6, 7, 14, 39, 40).

In the largest database analysis of 228 patients with SDC treated between 1973 and 2008, lymph node involvement, age, large tumor size, and tumor grade were associated with worse disease-specific survival (median OS was 79 months) (6). In another large national registry study in the Netherlands, OS, DFS, and MFS were 51, 23, and 26 months. Herein, the majority of patients (68%) initially presented with lymph node involvement, which is in line with our findings that greater boost volumes and nodal involvement were associated with inferior DFS and OS (14).

Clinical outcomes of 141 patients of a multi-institutional study cohort in Japan, where 59% of patients underwent postoperative radiotherapy, revealed that N+ was associated with lower OS and that the most common treatment failure was distant metastases in 39% (39). These results are consistent with the current study and underline the urgent need for improved systemic therapy.

A histopathological review of 75 cases, with the majority (81.3%) of patients receiving (chemo)radiotherapy, showed that PNI, LVI, and/or extracapsular spread were negative prognostic factors. The addition of chemotherapy to radiotherapy did not improve outcomes (40).

Additionally, there is no consensus on the role of systemic therapy in SDC in general (54–56). However, androgen receptors are found in 80 to 90% of SDC, as well as 30 to 70% expressing the human epidermal growth factor receptor (EGFR) and Her2neu, making the tumor a target for androgen deprivation therapy and monoclonal antibodies like cetuximab or trastuzumab, respectively (14, 40, 57–61). Recently, adjuvant androgen deprivation in patients with androgen receptor positive SDC has been shown do have a positive impact on DFS and seems to influence OS (62). In a histopathologic study of 50 SDC cases, expression of Her2neu was associated with a more aggressive course of disease (7). In this study, a significant proportion of the assessed tumors were positive for Her2neu and a subset received non-standardized trastuzumab as adjuvant treatment. Furthermore, the majority of tumors assessed were positive for androgen receptors, and received bicalutamide. However, the treatment period, intervals, and combinations thereof were extremely heterogenous, likely why no effect of any systemic therapy in the current analysis could be shown. Moreover, 50 to 70% of tumors expressing the EGFR-receptor may show benefit to EGFR-targeted therapy (56, 61, 63, 64). The high tendency for aggressive growth and patterns of failure demand the optimization of adjuvant treatment regimens. However, prospective trials remain elusive due to the rarity of the disease, even in a multicenter setting. A detailed list of series on surgical treatment and radiotherapy for SDC is provided in Table 2.

**Table 2:** Overview of the literature regarding management of salivary duct carcinoma.

**Authors/Study**	**Year**	**Sample size (number of patients)**	**Median time of follow-up (months)**	**Local control (%)**	**Received surgery (number of patients)**	**Received radiotherapy (number of patients)**	**Node positive tumors (number of tumors)**	**Results**
Afzelius et al. (27)	1987	12	NR	NR	12	12	5	Average survival: 21.7 months DOD: 7/12 (58%)
Brandwein et al. (28)	1990	12	NR	NR	12	6	8	DOD: 45% (5/11) within 10 years
Delgado et al. (29)	1993	15	NR	NR	15	9	10	DOD: 53% (8/15)
Kumar et al. (30)	1993	11	NR	NR	11	10	3	NR
Barnes et al. (31)	1994	13	24 (for 12/13)	92	13	5	7	DOD: 23% (3/13)
Grenko et al. (32)	1995	12	NR	NR	12	8	8	DOD: 33% (4/12) Median 12.5 months
Lewis et al. (9)	1996	26	NR	65	25	15	17	DOD within 3 years: 77% (20/26) Mean survival: 36 months 2-year survival: 58% 5-year survival: 30%
Guzzo et al. (15)	1997	26	36	64	25	18	15	2-year survival: 43% 5-year survival: 11.5%
Hosal et al. (33)	2003	15	34	79	15	14	11	DOD: 57% (8/14) Mean time to recurrence: 17 months
Jaehne et al. (7)	2005	50	NR	52	49	36	28	Average OS: 56.2 months Average time from first treatment to local recurrence: 17.4 months DOD: 56% (28/50) 5-year survival rate stage I: 42% 5-year survival rate stage II: 40% 5-year survival rate stage III: 30.8% 5-year survival rate stage IV: 23.2%
Kim et al. (20)	2012	35	48	63 (5-year)	35	35	26	Cause-specific death rate: 31.4% 5-year survival: 55.1% 5-year DFS: 47.4%
Shinoto et al. (19)	2013	25	44 (for 14/25)	67 (5-year)	25	25	15	5-year DFS: 45% 5-year survival: 47%
Jayaprakash et al. (6)	2014	228	53 (for survivors)	NR	223	166	111	DOD: 30% (70/228) after 10 years Median OS: 79 months 5-year DSS: 64% 10-year DSS: 56%
Shi et al. (34)	2014	38	39	NR	30	14	14	5-year DSS: 45% 5-year RFS: 30%
Roh et al. (35)	2014	56	71	87	44	47	40	Median DMFS: 36 months Median DSS: 48 months Median OS: 48 months
								Median OS: 48 months Median PFS: 16 months 5-year DMFS rate: 36% 5-year DSS rate: 44% 5-year OS rate: 42% 5-year PFS rate: 29%
Nakashima et al. (36)	2015	26	31	NR	26	19	20	3-year OS rate: 54% 5-year OS rate: 48.1%
Huang et al. (10)	2015	11	NR	NR	11	8	6	Mean OS time: 72.8 months 2-year OS rate: 75%
Schmitt et al. (37)	2015	28	NR	NR	28	11	20	Median DFS: 3.24 years Median OS: 4.65 years 5-year DFS: 49.2% 5-year OS: 49.3%
Luk et al. (8)	2016	23	26	NR	23	22	14	DOD: 43% (10/23) 5-year DFS: 36% 5-year DSS: 43%
Johnston et al. (38)	2016	54	68	83 (5-year)	53	49	44	5-year distant control: 48% 5-year OS: 43%
Otsuka et al. (39)	2016	141	36	90	134	83	71	3-year DFS: 38.2 % 3-year OS: 70.5%
Gilbert et al. (40)	2016	75	55	NR	71	61	54	Median DFS: 2.7 years Median OS: 3.1 years
Mifsud et al. (41)	2016	17	37	NR	17	17	13	Median OS: 49 months 3-year OS: 35.5% 3-year RFS: 34.4%
Breinholt et al. (11)	2016	34	28	NR	31	26	20	5-year DSS: 42% 5-year OS: 32% 5-year RFS: 35%
Haderlein et al. (18)	2017	67	26	NR	45	38	33	5-year DFS: 58.1% 5-year DMFS: 65.2% 5-year OS: 56.9%
Beck et al. (42)	2018	15	NR	100	15	14	9	2-year OS: 93%
Boon et al. (14)	2018	177	26	NR	162	149	120	Median DFS: 23 months Median DMFS: 26 months Median OS: 51 months
Anwer et al. (43)	2018	12	12	NR	11	10	3	10-month DFS: 75% 20-month DFS: 25%
Current studyAdeberg et al.	2019	28	30	96	23	28	18	Median DFS: 27 months Median DMFS: 69 months Median OS: 93 months Grad 3 toxicity: 21%

*DOD, dead of disease; NR, not reported; OS, overall survival; DFS, disease-free survival; DSS, disease-specific survival; RFS, recurrence-free survival; DMFS, distant metastasis-free survival*.

Toxicities herein were acceptable. In a retrospective analysis of patients with minor MSGT, several higher-grade toxicities were described, including dysphagia, xerostomia and also hearing loss, which were influenced by the target volume (65). Schulz-Ertner et al. described severe toxicity rates under 5% if radiotherapy is performed with modern techniques like IMRT combined with CIRT (66). Data of high-LET radiotherapy with neutrons produce late toxicities in approximately 10% (67), which is higher compared to these data, although follow up was relatively short herein. Furthermore, the retrospective design and the small patient sizes may add additional biases. In addition, adjuvant therapies were non-standardized and unmonitored herein. Despite these limitations, this is the first study to evaluate advanced radiation techniques using high-LET radiotherapy in SDC. Overall, the combination of surgical resection with neck dissection followed by dose-escalated radiotherapy with IMRT and CIRT leads to good LC. However, the high rate of distant metastases requires optimization of systemic therapies.

## Conclusions

Overall, the combination of surgical resection with neck dissection followed by dose-escalated radiotherapy with IMRT and CIRT leads to good local control rates. Larger tumor size and nodal involvement were associated with inferior disease control and survival. However, the limiting factor in patients with SDC is the high rate of distant mestastases, which is why adjuvant therapy need to optimized.

## Data Availability Statement

All datasets generated for this study are included in the article.

## Ethics Statement

The study was conducted in accordance with the Declaration of Helsinki. The study was approved by the ethics committee University Heidelberg (S-421/2015). Due to the retrospective nature of the evaluation of the performed standard therapy and the sole use of anonymized data, no study specific informed consent was necessary according to the local ethical guidelines.

## Author Contributions

SAd and PW: conceptualization. SAd, PW, and FE: methodology. SAd: formal analysis. SAd, PW, SAk, MB, TH, and DB: investigation. SAd and FE: writing—original draft preparation. SAd, JD, and SR: supervision. All authors: writing—review and editing.

### Conflict of Interest

SAd and DB received grants from Accuray International Sàrl outside the submitted work. DB received grants from Novocure outside the submitted work. JD received grants from CRI–The Clinical Research Institue GmbH, View Ray Inc., Accuray International Sàrl, Accuray Incorporated, RaySearch Laboratories AB, Vision RT limited, Merck Serono GmbH, Astellas Pharma GmbH, Astra Zeneca GmbH, Solution Akademie GmbH, Ergomed PLC Surrey Research Park, Siemens Healthcare GmbH, Quintiles GmbH, Pharmaceutecal Research Associates GmbH, Boehringer Ingelheim Pharma GmbH Co, PTW-Freiburg Dr. Pychlau GmbH, Nanobiotix AA outside the submitted work. The remaining authors declare that the research was conducted in the absence of any commercial or financial relationships that could be construed as a potential conflict of interest.
